# PlantReg 1.1 identifies the mutual arrangement of transcription factor binding sites in the target promoters for the elucidation of molecular mechanisms within regulatory networks

**DOI:** 10.18699/vjgb-25-100

**Published:** 2025-12

**Authors:** V.V. Lavrekha, N.A. Omelyanchuk, A.G. Bogomolov, Y.A. Ryabov, P.K. Mukebenova, E.V. Zemlyanskaya

**Affiliations:** Institute of Cytology and Genetics of the Siberian Branch of the Russian Academy of Sciences, Novosibirsk, Russia Novosibirsk State University, Novosibirsk, Russia; Institute of Cytology and Genetics of the Siberian Branch of the Russian Academy of Sciences, Novosibirsk, Russia; Institute of Cytology and Genetics of the Siberian Branch of the Russian Academy of Sciences, Novosibirsk, Russia; Institute of Cytology and Genetics of the Siberian Branch of the Russian Academy of Sciences, Novosibirsk, Russia; Institute of Cytology and Genetics of the Siberian Branch of the Russian Academy of Sciences, Novosibirsk, Russia Novosibirsk State University, Novosibirsk, Russia; Institute of Cytology and Genetics of the Siberian Branch of the Russian Academy of Sciences, Novosibirsk, Russia Novosibirsk State University, Novosibirsk, Russia

**Keywords:** gene ontology, biological processes, gene regulatory networks, binding site, transcription factor, Arabidopsis thaliana, генная онтология, биологические процессы, генные регуляторные сети, сайт связывания, транскрипционный фактор, Arabidopsis thaliana

## Abstract

The development of high-throughput sequencing has expanded the possibilities for studying the regulation of gene expression, including the reconstruction of gene regulatory networks and transcription factor regulatory networks (TFRNs). Identifying the molecular aspects for regulation of biological processes via these networks remains a challenge. Solving this problem for plants will significantly advance the understanding of the mechanisms shaping agronomically important traits. Previously, we developed the PlantReg program to reconstruct the transcriptional regulation of biological processes in the model species Arabidopsis thaliana L. The links established by this program between TFRNs and the genes regulating biological processes specify the type of regulation (activation/suppression). However, the program does not determine whether activation/suppression of the target gene is due to the cooperative or competitive interaction of transcription factors (TFs). We assumed that using information on the mutual arrangement of TF binding sites (BSs) in the target gene promoter as well as data on the activity type of TF effector domains would help to identify the cooperative/competitive action of TFs. We improved the program and created PlantReg 1.1, which enables precise localization of TF BSs in extended TF binding regions identified from genome-wide DAP-seq profiles (https://plamorph.sysbio.ru/fannotf/). To demonstrate the capabilities of the program, we used it to investigate the regulation of target genes in previously reconstructed TFRNs for auxin response and early reaction to salt stress in A. thaliana. The study focused on genes encoding proteins involved in chlorophyll and lignin biosynthesis, ribosome biogenesis, and abscisic acid (ABA) signaling. We revealed that the frequency of competitive regulation under the influence of auxin or salt stress could be quite high (approximately 30 %). We demonstrated that competition between bZIP family TFs for common BS is a significant mechanism of transcriptional repression in response to auxin, and that auxin and salt stress can engage common competitive regulatory mechanisms to modulate the expression of some genes in the ABA signaling pathway.

## Abstract (ru)

Развитие высокопроизводительного секвенирования расширило возможности изучения регуляции экспрессии генов, в том числе для реконструкции генных регуляторных сетей и регуляторных сетей транскрипционных факторов (РСТФ). Актуальной задачей остается выявление молекулярных аспектов регуляции данными сетями биологических процессов. Решение этой задачи для растений позволит существенно продвинуться в понимании механизмов формирования хозяйственно важных признаков. Ранее мы разработали программу PlantReg для реконструкции транскрипционной регуляции биологических процессов у модельного вида Arabidopsis thaliana L. Воспроизводимые этой программой связи между РСТФ и генами, обеспечивающими протекание биологических процессов, охарактеризованы по типу регуляции (активация/подавление). Однако программа не позволяла определять, в каких случаях активация/подавление экспрессии гена-мишени обусловлены кооперативным или конкурентным взаимодействием транскрипционных факторов (ТФ). Мы предложили использовать информацию о взаимном расположении сайтов связывания (СС) ТФ в промоторе гена-мишени, а также данные о типе активности трансактивационных доменов ТФ для выявления кооперативного/конкурент ного действия ТФ. Мы усовершенствовали программу, создав версию PlantReg 1.1, где обеспечили возможность точной локализации СС ТФ в протяженных районах связывания ТФ, устанавливаемых на основании полногеномных профилей DAP-seq (https://plamorph.sysbio.ru/fannotf/). Для демонстрации возможностей программы была исследована регуляция генов-мишеней ранее реконструированных нами РСТФ ответа на ауксин и солевой стресс у A. thaliana. В фокусе изучения были гены, кодирующие белки, участвующие в процессах биосинтеза хлорофилла и лигнина, биогенеза рибосом и в передаче сигнала абсцизовой кислоты. В данной работе установлено, что частота случаев конкурентной регуляции под влиянием ауксина и солевого стресса может быть достаточно высока (около 30 %). Показано, что конкуренция ТФ семейства bZIP за общие СС является значимым механизмом подавления транскрипции в ответ на ауксин, и что ауксин и солевой стресс могут задействовать общие механизмы конкурентной регуляции для модуляции экспрессии некоторых генов сигнального пути абсцизовой кислоты.

## Introduction

Development of genome-wide analysis techniques (such as RNA-seq (Deshpande et al., 2023), ChIP-seq (Park, 2009), and DAP-seq (O’Malley et al., 2016)) has opened up wide opportunities for systems biological research on mechanisms that ensure transcriptional regulation of biological processes and the formation of phenotypes (Marshall-Colón, Kliebenstein, 2019; Zemlyanskaya et al., 2021). Based on the analysis of genomic and transcriptomic data, the community is actively developing approaches to infer gene regulatory networks and TFRNs (Ko, Brandizzi, 2020; Rybakov et al., 2024). A TFRN is a set of regulatory interactions (links) between TF-coding genes, represented as a graph. The graph nodes correspond to the genes, and the directed edges reflect the regulatory interactions of a TF, encoded by one gene, with another gene. TFRN inference and identification of relationships between these networks and biological processes (or phenotypes) are essential to understanding the core regulatory circuits that drive biological processes, and to developing predictive models for these regulations (Huang et al., 2025; Leong et al., 2025; Sun Y. et al., 2025).

Several software tools for TFRN inference in various species are currently available to researchers. For example, the NetAct R package (Su et al., 2022) allows reconstructing mammalian TFRNs based on transcriptomic data and a database of TF target genes curated by the authors. Previously, we developed the CisCross-FindTFnet program for TFRN inference in the model plant species Arabidopsis thaliana (Omelyanchuk et al., 2024) and the PlantReg program for establishing regulatory links between TFRNs and genes that mediate the biological processes under the TFRN control (Lavrekha et al., 2024). Both programs integrate data from transcriptomic experiments and a representative collection of genome-wide DAP-seq TF binding profiles, with PlantReg employing the results of CisCross-FindTFnet as input data.

An important step in TFRN inference is to determine the mode of regulation exerted by a TF within the network (activators or repressors), since this characteristic shapes the network topology and dynamics (Dhatterwal et al., 2024). Large-scale determination of the activity of transcriptional effector domains in more than 400 A. thaliana TFs (Hummel et al., 2023) contributed to solving this problem. However, this is not sufficient for the correct classification of links within the network, since many TFs can function both as activators and suppressors, depending on the cell type, conditions, TF isoforms, specific promoters, and other factors (Boyle, Després, 2010; Martínez et al., 2018; Nagahage et al., 2018; Wang et al., 2020). This is why, when reconstructing the TFRN from transcriptomic data, the modes of regulation exerted by TFs are usually inferred from the profiles of their targets among differentially expressed genes (DEGs) (Su et al., 2022; Omelyanchuk et al., 2024).

Previously, we reconstructed two TFRNs in A. thaliana: the first, TFRN-A, controls the transcriptional response to auxin, the second, TFRN-S, controls the early response to salt stress (Lavrekha et al., 2024; Omelyanchuk et al., 2024). Using the PlantReg algorithm, we demonstrated how TFRN-A is involved in regulation of four different biological processes by auxin (activation of ribosome biogenesis and suppression of response to ABA, as well as chlorophyll and lignin biosynthesis), and how TFRN-S enhances ABA response during early salt stress. In these networks, TFs were divided into four classes: upregulated activator (UA), upregulated suppressor (US), downregulated activator (DA), and downregulated suppressor (DS). DAs and DSs form an R subnetwork (normally active before stimulus application, repressed due to stimulus action), UAs and USs set up an A subnetwork (activated by the stimulus).

**Fig. 1.**
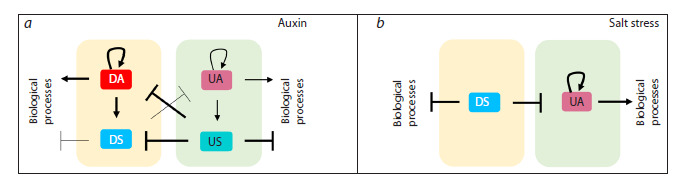
Principles of regulation of biological processes by TFs from TFRN-A (a) and TFRN-S (b). Yellow and green rectangles represent the repressed and activated subnetworks of TFRNs. Arrow thickness reflects the number of corresponding links in TFRNs. UA – upregulated activator; US – upregulated suppressor; DA – downregulated activator; DS – downregulated suppressor.

An important role of transcriptional repression has been identified in transcriptional responses to both auxin and salt stress. The auxin response is characterized by extensive reprogramming of the large R subnetwork, which was active before hormone treatment, through its suppression by US-type TFs from the A subnetwork (Fig. 1a) (Omelyanchuk et al., 2024). In contrast, the salt stress response activates the wide A subnetwork, partly through the inhibition of its DS-type suppressors from the R subnetwork (Fig. 1b) (Lavrekha et al., 2024).

The majority of the suppressors from both TFRNs are also involved in the regulation of the above-mentioned biological processes, affected by auxin and salt stress (Lavrekha et al., 2024; Omelyanchuk et al., 2024). However, according to the literature, most of the predicted suppressors in both TFRNs possess an activator-type transcriptional effector domain (Hummel et al., 2023; Omelyanchuk et al., 2024). Suppression of targets by these TFs may occur due to their cooperative or competitive interactions with other TFs. The PlantReg program enables establishing regulatory links between TFs and genes that mediate biological processes, but it does not detect cooperation or competition among TFs. At the same time, it is crucial to understand the mechanisms of TF interactions in transcriptional regulation to effectively use TFRNs and their relations to biological processes in plant bioengineering.

**Fig. 2.**
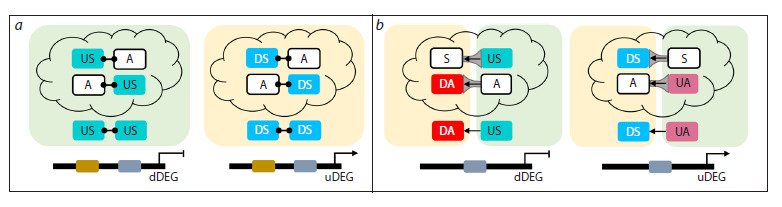
Cooperative (a) and competitive (b) regulation of a target gene by a pair of TFs from a TFRN. Yellow and green rectangles represent the repressed and activated TFRN subnetworks. Predicted TF modes of regulation are shown at the bottom, while possible alternative modes are shown at the top (in the cloud). The connected dots between TFs in (a) denote protein interactions; in (b) arrows represent the substitution of one TF with another after stimulus application and gray funnels designate the ratio of TF activities (larger bases correspond to higher activity); uDEG – upregulated DEGs; dDEG – downregulated DEGs; UA – upregulated activator; US – upregulated suppressor; DA – downregulated activator; DS – downregulated suppressor

Information on the mutual arrangement of the TF binding sites (BSs) in the target promoter, coupled with data on the activity of the TF effector domains (Hummel et al., 2023), can be used to identify and characterize the cooperative or competitive action of TFs. For example, if the BSs of two predicted suppressors, operating within the same subnetwork, are close to each other, and only one has a transcriptional effector domain exhibiting suppressor activity, while the other TF is a transcriptional activator, it is plausible to assume that a cooperative interaction between TFs converts an activator TF into a repressor (Fig. 2a). Such examples are widespread and described in detail in the literature (Hanna-Rose, Hansen, 1996; Ahn et al., 2006; Veerabagu et al., 2014; Martínez et al., 2018; Wang et al., 2020).

Similarly, if the BSs of a predicted activator from one subnetwork and a predicted repressor from another subnetwork overlap in the promoter of a target gene, and the predicted activity of one of the TFs does not match the established activity of its transcriptional effector domain, we can assume that TFs may compete for the common BS, and the replacement of a strong activator with a weaker one manifests itself as suppression of the target gene, while the replacement of a strong repressor with a weaker one manifests itself as activation of the target gene (Fig. 2b). A decrease in promoter activity with an increase in the concentration of a weak activator compared to a strong one, as well as the reverse transition, have been shown in a number of experiments (Tamura et al., 2004; Zhang et al., 2006; Chupreta et al., 2007; Selvaraj et al., 2015; Ren et al., 2015; Brackmann et al., 2018).

To identify TF targets, the PlantReg program recruits DAPseq peaks. However, this does not enable precise localization of TF BSs, since the peak size (over 150 bp) significantly exceeds the length of the sequences recognized by TFs (below 20 bp). In this study, we improved the program by creating PlantReg version 1.1, which enables precise localization of TF BSs in extended TF binding regions from genome-wide DAP-seq profiles (https://plamorph.sysbio.ru/fannotf/). We used PlantReg 1.1 to identify genes involved in chlorophyll and lignin biosynthesis, ribosome biogenesis, and ABA signaling, the expression of which can be suppressed under TFRN-A or TFRN-S control due to competition between TF activators for common BSs.

The analysis revealed that the frequency of competitive regulation under auxin and salt stress exposure can be quite high. Furthermore, we demonstrated that competition between bZIP family TFs for common BSs is an essential mechanism for transcription repression in A. thaliana auxin response, and that auxin and salt stress can utilize common competitive regulation to modulate the expression of some genes in ABA signaling.

## Materials and methods

**Fig. 3.**
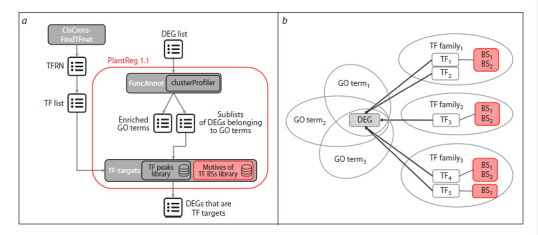
PlantReg 1.1 workflow (a) and output structure (block 1) (b). Updates in PlantReg 1.1 compared to the original version are highlighted in pink.

**Integration of data on TF BSs in 5′-regulatory regions into PlantReg 1.1.** The original PlantReg version (Lavrekha et al., 2024) was designed to reconstruct the mechanisms underlying transcriptional regulation of biological processes in A. thaliana based on the analysis of a DEG list and a list of TFs – known or putative transcriptional regulators of these DEGs. PlantReg performs gene ontology (GO) enrichment analysis of the input DEG list, and identifies potential TF targets among DEGs associated with enriched biological processes, recruiting genome-wide TF binding profiles available in the web version of the program (Fig. 3a). The output of PlantReg is presented in five blocks, which reflect the relationships between biological processes, DEGs, and TFs that regulate the expression of these DEGs.

The basic workflow of the updated PlantReg 1.1 version is shown in Figure 3. In addition to the original functionality, it includes data on recognized TF BSs in the 5′-regulatory regions (Fig. 3a), which are added to output blocks 1 and 4 to enable investigation on the mutual arrangement of BSs in promoters. The output block 1 in the original PlantReg version presents a sublist of DEGs associated with enriched biological processes (Fig. 3b). Each gene in the sublist is characterized by a set of associated GO terms (biological processes) with evidence codes, the number of GO terms, a list of potential transcriptional regulators with an indication of their TF families, and the number of TFs.

In output block 4, the same information is presented in an alternative format with the GO terms and transcriptional regulators for each gene listed line by line. In PlantReg 1.1, the nucleotide sequence of the TF BS recognized in the corresponding TF binding region, the genomic coordinates of the TF BS (block 4) or the coordinates of the TF BS relative to the transcription start site (block 1), and the DNA strand harboring the TF BS were added to the description of each gene (Fig. 3b). Information on the TF BS localization is available only when the CisCross-MACS2 genome-wide profile collection is selected as a parameter.

**Recognition of TF BSs in the 5′-regulatory regions of A. thaliana genes.** Position frequency matrices describing the BSs of A. thaliana TFs were generated by de novo motif search in DAP-seq peaks from the CisCross-MACS2 collection available in the web version of the PlantReg program (Lavrekha et al., 2024). The CisCross-MACS2 peak set collection was compiled previously (Lavrekha et al., 2022) by processing raw data from genome-wide DAP-seq profiling of BSs for 403 A. thaliana TFs (O’Malley et al., 2016). In each peak set, the top 2,000 peaks were selected by height and used for de novo motif search employing the STREME program (Bailey et al., 2021).

A background set was generated by the AntiNoise program (Raditsa et al., 2024). The motif with the highest enrichment significance (with a p-value below 0.05) was assumed to describe the BS for TF of interest. To test this assumption, the identified motifs were juxtaposed to known TF BSs by comparing with motifs from the JASPAR2024 CORE (Rauluseviciute et al., 2024), CisBP (Weirauch et al., 2014), and ArabidopsisDAPv1 (O’Malley et al., 2016) databases using the Tomtom program (Gupta et al., 2007).

The search for potential TF BSs in the 5′-regulatory regions of A. thaliana genes ([–2500; +1) relative to the transcription start site) was performed using the position weight matrix method with the scan_sequence function of the universalmotif R-package (Tremblay, 2024). To extract the nucleotide sequences of the 5′-regulatory regions, the A. thaliana TAIR10 genome version (Lamesch et al., 2012) and the Araport11 genomic annotation (Cheng et al., 2017) were used.

**Fig. 4.**
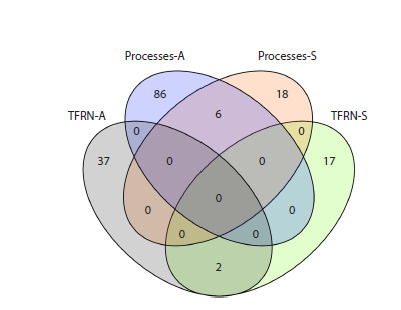
The number of genes encoding TFs in TFRN-A and TFRN-S, as well as TF target genes, which mediate biological processes affected by auxin (Processes-A) or salt stress (Processes-S). The “Processes-A” set includes genes for chlorophyll biosynthesis, lignin biosynthesis, ribosome biogenesis and ABA transport, conjugation, and the signaling pathways. The “Processes-S” set includes only genes for ABA transport, conjugation, and the signaling pathway.


**Search for genes, the transcription of which is regulated by competitive suppression or activation. **Regulatory links between components of the TFRN-A/S (Lavrekha et al., 2024; Omelyanchuk et al., 2024) and genes involved in biological processes affected by auxin and salt stress, as well as competitive gene suppression or activation under auxin and salt stress exposure were identified using PlantReg 1.1. As input, we used the lists of TFs that constituted TFRN-A (39 elements) and TFRN-S (19 elements) (the lists are designated as “TFRN-A” and “TFRN-S” in Fig. 4) (Table S1)1, as well as the lists of DEGs upregulated (uDEGs) and downregulated (dDEGs) by auxin (5,201 uDEGs and 6,704 dDEGs) or salt stress (1,476 uDEGs and 944 dDEGs), which were used previously to reconstruct the TFRNs (De Rybel et al., 2012; Wu et al., 2021; Omelyanchuk et al., 2024).

Supplementary Materials are available in the online version of the paper: https://vavilov.elpub.ru/jour/manager/files/Suppl_Lavrekha_Engl_29_7.xlsx


The lists of uDEGs and dDEGs from the two transcriptomic experiments were separately fed into the PlantReg 1.1 program along with the corresponding list of TFs from the TFRN-A or TFRN-S. The threshold for GO terms enrichment was set at 0.001. To localize the TF binding regions, the CisCross- MACS2 collection of genome-wide TF binding profiles and a 5′-regulatory region length of 1,000 bp were selected. This analysis resulted in “TF-regulator–target gene” pairs, where the TFs from the TFRN-A or TFRN-S were TF-regulators, and the uDEGs and dDEGs from the corresponding transcriptomic experiment were the target genes.

The DAP-seq data, recruited by PlantReg 1.1 to map TF binding regions in the A. thaliana genome, contain two types of peak sets: in the first case (“col” peak sets), native genomic DNA from leaves was used to prepare libraries; in the second case (“colamp” peak sets), genomic DNA with methylcytosine epigenetic marks removed by PCR amplification was used. TF-regulator–target gene pairs reconstructed using “col” peak sets were selected from the PlantReg 1.1 output. Next, among the target genes regulated by TFRN-A, we chose the genes annotated with GO terms related to chlorophyll biosynthesis (16 genes), lignin biosynthesis (14 genes), ABA signaling (34 genes), and ribosome biogenesis (28 genes); these processes were previously considered in (Omelyanchuk et al., 2024).

Among the target genes regulated by TFRN-S, we selected genes annotated with GO terms related to ABA signaling (24 genes), which was previously discussed in (Lavrekha et al., 2024). As a result, 110 genes were chosen (designated as “Processes-A” and “Processes-S” in Fig. 4) (Tables S1–S3).

To identify among these genes the ones potentially regulated by competitive suppression or activation, we selected the genes that met the following requirements: a) more than one TF was involved in the regulation of the gene, b) the BSs of these TFs considerably overlapped (over 80 %), and c) the genes encoding these TFs changed their expression in opposite directions in the transcriptomic experiment.

## Results

**A collection of the predicted TF BSs in 5’-regulatory regions of A. thaliana genes, integrated into PlantReg 1.1**

**Fig. 5.**
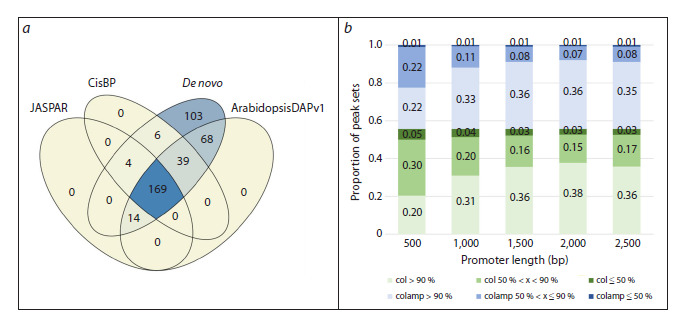
Characteristics of the collection of predicted TF BSs in the 5’-regulatory regions of A. thaliana genes integrated into PlantReg 1.1 a – comparison of motifs recognized de novo in DAP-seq peaks with known TF BSs in the JASPAR, CisBP, and ArabidopsisDAPv1 databases; b – proportions of DAP-seq peak sets mapped to the 5’-regulatory regions (col – shades of green/colamp – shades of blue) with the motifs recognized in more than 90 % of peaks (light shade), in 50–90 % of peaks, and in less than 50 % of peaks (dark shade).

To enable prediction of cooperative and competitive interactions of TFs in the transcriptional regulation of biological processes, automatic localization of TF BSs in 5′-regulatory regions was implemented in PlantReg 1.1. For this purpose, the results of TF BS recognition in promoters using the position weight matrices (see the “Materials and methods” section) were systematized and integrated into PlantReg 1.1. For 300 TFs (74 %), the motif identified de novo in at least one peak set (“col” or “colamp”) was similar to a known BS for this TF available in the JASPAR, CisBP, or ArabidopsisDAPv1 databases (Fig. 5a). The proportion of TFs with BSs recognized in more than 90 % of peaks mapped to 5′-regulatory regions was quite high and varied from 42 (for 500 bp-long 5′-regulatory regions) to 74 % (for 2,000 bp-long 5′-regulatory regions) (Fig. 5b).

In the following sections, we illustrate the potential of using the new functionality of PlantReg 1.1 to solve specific biological challenges.

**Competitive regulation of gene expression in response to auxin and salt stress in A. thaliana**

We assumed above that the suppression of target gene transcription with an increase in the level of US-type TFs or activation due to a decrease in the level of DS-type TFs in response to auxin and under salt stress may occur through competitive regulation of their expression by a pair of activator TFs. To test this hypothesis, we identified regulatory links between TFRN-A/S and genes involved in chlorophyll and lignin biosynthesis, ribosome biogenesis, and ABA signaling using PlantReg 1.1. Fourteen genes were picked as potential targets for competitive regulation by TFs from TFRN-A and TFRN-S (Tables S1, S6 and S7).

Additionally, 11 genes encoding TFs from TFRN-A and TFRN-S were also found as potential targets for competitive regulation (Tables S4 and S5). All 25 selected genes (12 dDEGs, 10 uDEGs, and three genes, ABCG25 (ATPbinding cassette family G25), GBF3 (G-box binding factor 3), and PYL7/RCAR2 (PYR1-like 7/Regulatory components of ABA receptor 2), the expression of which changed in opposite directions under auxin and salt stress) made up as much as 32 % of the total number of genes regulated by suppressors (79 genes) (Tables S1, S6 and S7). Thus, the competitive regulation of the target genes by TFRNs may be a frequent event.

TFs are grouped into families, classes, and superclasses based on the similarity of their DNA-binding domains (Blanc-Mathieu et al., 2024). TFs from the same family often recognize similar DNA sequences and, therefore, can compete for the binding sites. In the 5′-regulatory regions of 25 selected genes, TFs can compete within the following six families: AP2/ERF (APETALA2/ETHYLENE RESPONSIVE FACTOR), bZIP (BASIC LEUCINE-ZIPPER), BZR1/BES1 (BRASSINAZOLE RESISTANT 1/BRI1 EMS SUPPRESSOR 1), HD-ZIP (HOMEODOMAIN LEUCINE ZIPPER), MYB (V-MYB AVIAN MYELOBLASTOSIS VIRAL ONCOGENE HOMOLOG), WRKY (Table S6). In addition, we identified possible competition between TFs from different families of the same superclass, namely: “basic domains” and “Helix-Turn-Helix domains” (Table S6).

Moreover, in the promoters of uDEGs MAPKKK18 (Mitogen- activated protein kinase kinase kinase 18) and RRP47 (Sas10/Utp3/C1D family), the same BS can be occupied by TFs from the families belonging to two different superclasses: AP2/ERF (“Beta-hairpin exposed by an alpha/beta-scaffold” superclass) and bZIP (“basic domains” superclass) in the first case, and AP2/ERF and LBD (“Zinc-coordinating DNA binding domains” superclass) in the second case (Table S6). In the distal promoter of dDEG GBF3, TFs from the families of two other superclasses, bZIP (“basic domains”) and MYB (“Helix-Turn-Helix domains”), can compete for the common BS. We also detected a possible competition for the common BS among TFs from different families belonging to two (in the promoters of AFP1 (ABI five binding protein), MYB73 and PYL7) and even three different superclasses (in the distal promoter of GBF3) (Table S6).

**Competition of bZIP family TFs in promoters of genes regulated by TFRN-A**

**Fig. 6.**
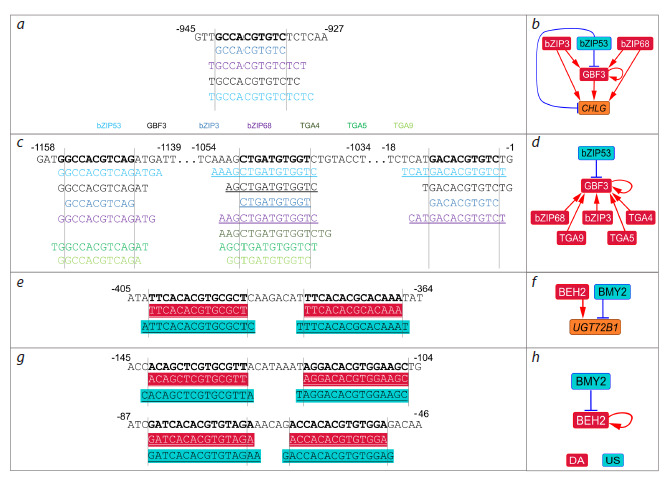
Overlapping TF BSs in target gene promoters revealed with PlantReg 1.1. а – distal CHLG promoter with overlapping BSs for bZIP family TFs (bZIP3, bZIP53, bZIP68, and GBF3); c – distal and core GBF3 promoters with overlapping BSs for bZIP family TFs (bZIP3, bZIP53, bZIP68, TGA4/5/9, and GBF3), TF color coding in (c) is the same as in (a); e, g – proximal UGT72B1 and BEH2 promoters, respectively, with overlapping BSs for BEH2 (red fill color) and BMY2 (blue fill color). b, d, f, h – transcriptional regulation of the CHLG, GBF3, UGT72B1, and BEH2 genes, respectively. Underlined BSs lie on the antisense strand with regard to the gene body strand. Coordinates are given relative to the transcription start site. US – upregulated suppressor; DA – downregulated activator

To identify combinations of activator TFs systematically recruited by TFRN-A or TFRN-S to suppress target gene expression, we conducted a comparative analysis of TF-regulator– target gene pairs determined with PlantReg 1.1. Three DA-type TFs (bZIP3, bZIP68, and GBF3) and a US-type TF (bZIP53) share common BSs in the promoters of several genes regulated by TFRN-A. These include CHLG (Chlorophyll G) (Fig. 6a, b), HEME2 (AT5G14220), and CH1 (Chlorina 1), which encode chlorophyll biosynthetic enzymes, as well as ABCG25, encoding ABA exporter that transports ABA across the plasma membrane (Tables S6 and S7).

Since bZIP53 was described in the literature as a transcriptional activator (Alonso et al., 2009; Weltmeier et al., 2009), it is logical to assume that the suppression of the above-mentioned genes may be a consequence of competition among bZIP family TFs for common BSs in promoters, resulting in replacement of a strong activator by a weaker one. Indeed, the activity of the transactivation domains of these TFs was previously investigated and it was shown that bZIP53 is a transcriptional activator, but a much weaker one than representatives of the same family bZIP3, bZIP68, and GBF3 (Hummel et al., 2023).

It is noteworthy that a similar combination of transcriptional regulators competing for a common BS (bZIP3, bZIP68, and GBF3 as DA, bZIP53 as US) was identified in the promoters of dDEGs ERF15, GBF3 (Fig. 6c, d), and AT1G19000 encoding TFs from the TFRN-A (Tables S6 and S7). Thus, competition between the bZIP family TFs for a common BS is likely to be an essential mechanism of transcriptional repression in auxin response.

We also found a potential replacement of the bZIP3, bZIP68, and GBF3 activators with a weaker one, bZIP53, in the promoter of GBF3, which itself encodes a TF involved in its competitive regulation (Fig. 6c, d). A similar situation was observed for BEH2 (BES1/BZR1 HOMOLOG2) (DA) and BMY2 (BETA-AMYLASE 2, also known as BETAAMYLASE 8/BAM8) (US), both belonging to the BZR1/ BES1 family. These TFs regulate not only the expression of DFB and UGT72B1, the genes that control lignin levels, but also BEH2 (Fig. 6e–h).

Theoretically, such feedback could act as a “trigger” for more intensive competitive suppression of common targets by a pair of activator TFs: an increase in the abundance of a weaker transcriptional activator leads to competitive suppression of the gene encoding a stronger transcriptional activator (which is a common target for both TFs including that stronger one), and thereby the inhibitory effect on other common targets will increase. Some DA-type TFs can compete with each other for a common BS prior to auxin treatment, when R subnetwork is active (Fig. 6a–d), providing additional regulatory flexibility to TFRN-A.

**Competitive regulation of ABA signaling genes by TFRN-A and TFRN-S**

Both auxin and salt stress modulate response to ABA: in the first case, it is attenuated, and in the second case, it is enhanced (Lavrekha et al., 2024; Omelyanchuk et al., 2024). Comparison of the regulatory links inferred based on data from different experiments enables a deeper understanding of transcription regulation. Using PlantReg 1.1, we found that three genes involved in ABA signaling (PYL7, AFP1, and ABCG25) are under the control of both TFRNs

**Fig. 7.**
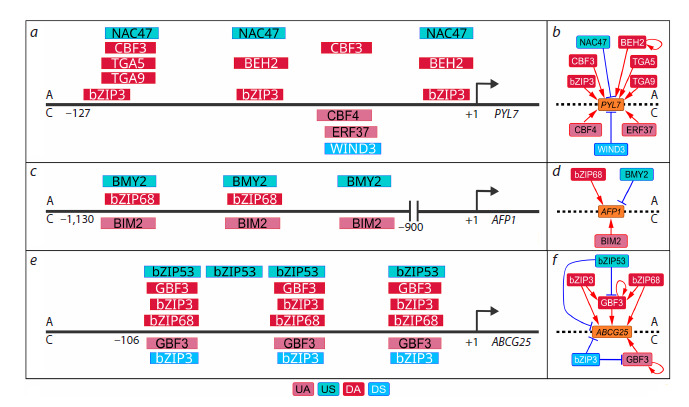
Overlap of TF BSs in target promoters under auxin treatment and early salt stress, revealed using PlantReg 1.1. a – proximal PYL7 promoter; c – distal AFP1 promoter; e – proximal ABCG25 promoter. b, d, f – transcriptional regulation of the PYL7, ATF1, and ABCG25 genes, respectively. For each panel, the details of regulation in response to auxin (A) and early salt stress (S) are located at the top and bottom, respectively. TF BSs are represented by rectangles according to the color coding of the regulation type: UA – upregulated activator; US – upregulated suppressor; DA – downregulated activator; DS – downregulated suppressor.

Downregulation of PYL7 by auxin and its upregulation by salt stress is carried out by TF sets specific for each stimulus. These TF sets bind to different sites in the PYL7 promoter (Fig. 7a, b). Apparently, auxin and salt stress utilize distinct molecular mechanisms for competitive modulation of PYL7 expression. In contrast, both stimuli can engage the same set of competing activator TFs to regulate AFP1 and ABCG25, but in different ways. AFP1 gene expression is mediated by bZIP68 under normal conditions. After auxin treatment, bZIP68 is replaced by BMY2 (which is likely a weaker activator); under salt stress, on the contrary, bZIP68 is replaced by BIM2 (BES1-interacting Myc-like protein 2), which is a stronger activator according to (Hummel et al., 2023) (Fig. 7c, d). In the ABCG25 promoter, auxin induces replacement of activator TFs from the bZIP family with a weak activator bZIP53 that results in a decrease in ABCG25 transcripts (Fig. 7e, f ). Salt stress modulates the relocation of a similar set of activators within the same set of BSs, but in this case, downregulation of bZIP3 expression is accompanied by accumulation of GBF3 transcripts.

**Fig. 8.**
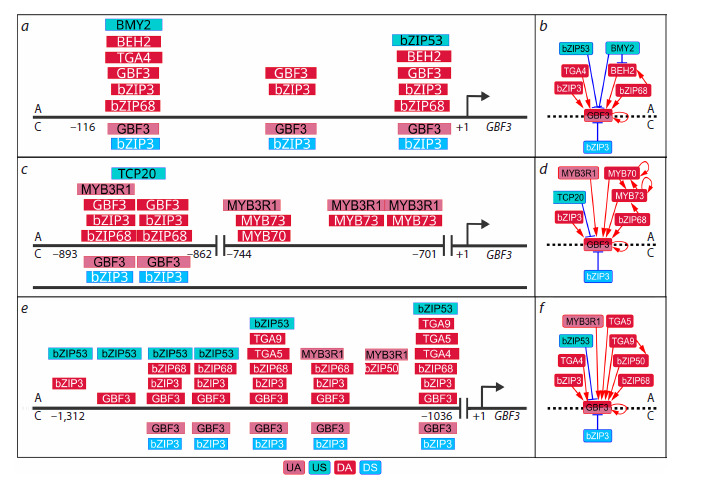
Overlapping BS TFs in the GBF3 promoter under auxin treatment and early salt stress, revealed using PlantReg 1.1. a, c, e – proximal (–116; +1) and distal (–1,312; –701) GBF3 promoters with overlapping BS TFs; b, d, f – diagrams of GBF3 transcriptional regulation in the proximal and distal promoters. For each panel, the regulations in response to auxin (A) and early salt stress (S) are located at the top and bottom, respectively. TF BSs are represented by rectangles according to the color coding of the regulator type: UA – upregulated activator, US – upregulated suppressor, DA – downregulated activator, DS – downregulated suppressor.

Interestingly, a similar pattern was observed in the promoter of GBF3 encoding a TF involved in both TFRNs. Under salt stress, which activates GBF3, GBF3 TF replaces bZIP3 at three BSs in the proximal GBF3 promoter (–116; +1) (Fig. 8a, b), and at seven BSs in the distal promoter (–1,312; –701) (Fig. 8c–f ), thereby apparently enhancing its self-activation. After auxin treatment, another redistribution of bZIP family TFs occurs at the same sites (Fig. 8). These results are consistent with an important role of competition for BSs between bZIP family TFs in modulation of gene expression (Schindler et al., 1992; Foster et al., 1994; Ko, Brandizzi, 2022). At the same time, auxin response recruits some specific mechanisms for GBF3 regulation that are not involved in the response to salt stress. Thus, MYB3R1 can replace MYB70 and MYB73 at the common site after auxin treatment.

## Discussion

In this work, we collected and systematized information on potential TF BSs in A. thaliana promoters to integrate it into the PlantReg 1.1 program. Along with the data on TF effector domain activity (Hummel et al., 2023), this allows to predict the cooperative and competitive interaction of TFs within the TFRNs in the transcriptional regulation of biological processes. Previously, we reconstructed two TFRNs that control the responses to salt stress and auxin in A. thaliana and showed that transcriptional repression plays an important role in both cases (Lavrekha et al., 2024; Omelyanchuk et al., 2024). However, according to the literature, the overwhelming majority of predicted suppressors in the TFRNs have activatortype effector domains (Hummel et al., 2023; Omelyanchuk et al., 2024). We used PlantReg 1.1 to identify the molecular mechanisms underlying the possible transformation of activator TFs into transcriptional repressors.

We found that more than one-third of the targets of TFs that were predicted as suppressors could be competitively regulated by a pair of TFs, one of which is a strong transcriptional activator and the other is a weak one. Thus, competitive regulation of gene expression is likely a universal mechanism allowing modulation of gene expression during responses to salt stress and auxin in A. thaliana.

Auxin is a key regulator of most plant processes involved in switching between developmental programs (Vanneste et al., 2025). The most standard concept of switching is replacement of a repressor with an activator, such as the substitution of the E2F TF repressor complex with E2F activators before the onset of the cell cycle in the promoters of many plant and animal genes (van den Heuvel, Dyson, 2008; Sánchez-Camargo et al., 2021), or, conversely, replacement of a transcriptional activator with a repressor (Berlow et al., 2017). However, the results obtained with PlantReg 1.1 indicate that in the auxin response, instead of the canonical activator–repressor switch, substitution of a strong activator with a weaker one can be actively used to suppress transcription.

At least some of the cases when a strong activator is substituted with a weaker one, predicted by PlantReg 1.1, are supported by previously published data. These include, for example, the replacement of three activators, bZIP3, bZIP68, and GBF3, by a weaker activator bZIP53 during auxin-induced suppression of chlorophyll biosynthesis genes CHLG, HEME2, and CH1 (Hummel et al., 2023). Competition between bZIP family TFs for a common binding site and its influence on target gene expression has been previously described for many TFs from this family (Schindler et al., 1992; Foster et al., 1994; Ko, Brandizzi, 2022). It is also known that a number of bZIP family TFs redundantly regulate chlorophyll biosynthesis in a complex manner. In particular, chlorophyll biosynthesis is impaired in the gbf1 gbf2 gbf3 triple mutant, demonstrating the important role of GBFs in this process (Sun T. et al., 2025). Overexpression of another family member, bZIP1, results in decreased chlorophyll levels, while the bzip1 bzip53 double mutant demonstrates a less pronounced decrease in chlorophyll levels and attenuated CHLG expression compared to the wild type ( padj = 0.03) (Wildenhain et al., 2025).

The plant-specific BZR1/BES1 TF family mediates transcriptional response to brassinosteroids (plant steroid hormones). In addition to BZR1 and BES1, this family also includes four other TFs, called BES1 homologues: BEH1, BEH2, BEH3, and BEH4 (Shi et al., 2022). Recently, the BZR1/BES1 family has been supplemented with two unusual TFs, BAM7 and BMY2, which are similar to β-amylases but also exhibit very weak homology to BES1 (Reinhold et al., 2011). These TFs lack amylase catalytic activity but possess BZR1-like domains that bind to the sequences recognized by TFs from this family. BMY2 is a transcriptional activator, while BAM7 regulates its activity.

It has been previously suggested that BMY2 controls the transcription of target genes by competing with the other BZR1/BES1 TFs for BSs (Reinhold et al., 2011). According to the results obtained with PlantReg 1.1, this may take place in the promoters of some genes downregulated by auxin, including UGT72B1 (UDP-glucose-dependent glucosyltransferase 72 B1), which encodes a monolignol-conjugating enzyme. In the UGT72B1 promoter, BMY2 (which is likely a weaker activator) competes with BEH2 (Fig. 6e, f; Tables S6 and S7).

A more detailed analysis of the BEH2 and BMY2 targets predicted using PlantReg 1.1 provides several important details to auxin regulation of lignin biosynthesis. Auxin, by activating BMY2, inhibits BEH2 self-activation replacing BEH2 with the less active BMY2 TF at their common BSs (Fig. 5g, h). This leads to a decrease in BEH2 levels in the nucleus, which in turn facilitates the replacement of this TF at its sites in the UGT72B1 promoter with a weaker activator BMY2 (Fig. 5e, f ) and, consequently, causes a decrease in the UGT72B1 transcript level. Activation of UGT72B1 by BMY2 is supported by an increase in UGT72B1 transcript level upon BMY2 overexpression and downregulation of this gene in the bmy2 bam7 double mutant (Reinhold et al., 2011).

Notably, auxin suppresses the transcription of most genes encoding lignin biosynthetic enzymes (Omelyanchuk et al., 2024), thereby reducing monolignol levels. At the same time, auxin downregulates UGT72B1 expression and as a consequence inhibits monolignol conjugation, partially compensating for the decrease in monolignol levels. Interestingly, brassinosteroids also modulate lignin levels through BEH2. Brassinosteroids enhance lignin biosynthesis by activating most of the enzymes involved in this process (Percio et al., 2025). They simultaneously suppress BEH2 via both GSK3 (GLYCOGEN SYNTHASE KINASE 3)-like kinases and BES1 (Otani et al., 2022). Since BEH2 activates UGT72B1, which conjugates monolignols, brassinosteroids restrict the withdrawal of monolignols from lignin biosynthesis, thereby further increasing the lignin level.

The data obtained using PlantReg 1.1 allow formulating specific hypotheses for planning further experimental studies. It is worth emphasizing, however, that these predictions may contain false-positive results. For example, in the pair of TFs HB21 (DA) and HB40 (US) from TFRN-A, which bind the same sites in the promoter of the auxin-repressed gene bZIP50, HB40 is a more potent activator. This means that competition for BS with HB21 cannot explain the suppression of target gene expression with HB40 increase. It is possible that HB21 and HB40 are expressed in different tissues or at different developmental stages. To explain why HB40, which is an activator by nature, can function as a repressor, we need to explore how this TF recruits corepressors

## Conclusion

PlantReg 1.1 is designed to reveal regulatory relationships between TFRNs and genes that mediate the biological processes controlled by these networks. The updated version of the program includes functionality for precise localization of TF BSs in target promoters. Due to this, it becomes possible to analyze the mutual arrangement of TF BSs and, using data on the effector TF domains, to identify potential cooperative or competitive TF action in the promoter of a particular gene.

PlantReg 1.1 was successfully applied to reconstruct the transcriptional mechanisms regulating chlorophyll and lignin biosynthesis, ribosome biogenesis, and ABA response under auxin and salt stress. Analysis of the mutual arrangement of TF BSs revealed that the activity of a number of genes regulating these processes can be suppressed as a result of competition between a pair of activator TFs for a common BS, with a weaker activator replacing a stronger one. Some of the obtained results were supported by literature data.

Thus, the results obtained using PlantReg 1.1 allow formulating specific hypotheses for planning further experimental studies. It is worth emphasizing, however, that the predictions may contain false-positive results. Reducing their incidence is one possible direction for further development of the program.

## Conflict of interest

The authors declare no conflict of interest.
